# Advancing Self‐Assembled Molecules Toward Interface‐Optimized Perovskite Solar Cells: from One to Two

**DOI:** 10.1002/adma.202502032

**Published:** 2025-04-29

**Authors:** Tanghao Liu, Chuanyao Luo, Ruiqin He, Zhuoqiong Zhang, Xiaohui Lin, Yimu Chen, Tom Wu

**Affiliations:** ^1^ School of Physical Sciences Great Bay University Dongguan Guangdong 523000 China; ^2^ Department of Applied Physics Hong Kong Polytechnic University Hong Kong Hong Kong SAR 999077 China; ^3^ Ministry of Industry and Information Technology Key Lab of Micro‐Nano Optoelectronic Information System Guangdong Provincial Key Laboratory of Semiconductor Optoelectronic Materials and Intelligent Photonic Systems School of Integrated Circuits Harbin Institute of Technology (Shenzhen) Shen Zhen Guangdong 518067 China

**Keywords:** aggregation, co‐SAM, energy level, perovskite, wettability

## Abstract

Perovskite solar cells (PSCs) have rapidly gained prominence as a leading candidate in the realm of solution‐processable third‐generation photovoltaic (PV) technologies. In the high‐efficiency inverted PSCs, self‐assembled monolayers (SAMs) are often used as hole‐selective layers (HSLs) due to the advantages of high transmittance, energy level matching, low non‐radiative recombination loss, and tunable surface properties. However, SAMs have been recognized to suffer from some shortcomings, such as incomplete coverage, weak bonding with substrate or perovskite, instability, and so on. The combination of different SAMs or so‐called co‐SAM is an effective strategy to overcome this challenge. In this Perspective, the latest achievements in molecule design, deposition method, working principle, and application of the co‐SAM are discussed. This comprehensive overview of milestones in this rapidly advancing research field, coupled with an in‐depth analysis of the improved interface properties using the co‐SAM approach, aims to offer valuable insights into the key design principles. Furthermore, the lessons learned will guide the future development of SAM‐based HSLs in perovskite‐based optoelectronic devices.

## Graphical abstract

In the past few years, self‐assembled molecules (SAMs) have ushered in a new era of interface engineering for perovskite solar cells. Herein, the recent progresses of co‐SAM, namely two SAMs with synergy, are comprehensively summarized and analyzed, focusing on topics including deposition methods and design principles, while further challenges about mechanisms, materials, and applications are also outlined.

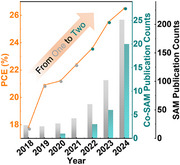

## 1 Introduction

In the past decade, perovskite solar cells (PSCs) have emerged as a promising candidate for next‐generation photovoltaic technology, featuring cost‐effective manufacture, tunable bandgap, high efficiency, and compatibility with tandem technologies.^[^ ^1^ ^]^ PSCs could be easily solution processed at a low temperature (<150 °C), which makes their fabrication cost much lower than that of traditional solar cells. Besides, their certified power conversion efficiency (PCE) has reached 27%, which is comparable with the record PCE of single crystalline silicon solar cells.^[^ ^2^ ^]^ Owing to such low cost and high efficiency, PSCs show great potential for commercialization. Nowadays, most PSCs adopt the planar configuration, in which a perovskite layer is sandwiched by a hole selective layer (HSL) and an electron selective layer (ESL). Charge selective layers (HSLs, ESLs) are responsible for facilitating the charge carrier separation and extraction. The ideal HSL/ESL should contact the perovskite layer with a conformal interface for the minimum current leakage, and good energy‐level alignment for efficiency charge extraction. Besides, HSL/ESL is expected to not react with perovskite or accelerate the degradation of perovskite. According to the deposition sequential, the device structures are divided into regular (n‐i‐p) and inverted (p‐i‐n) structures.^[^ ^3^, ^4^ ^]^ In regular PSCs, the widely used HSL, 2,2′,7,7′‐tetrakis(N, N‐di‐p‐methoxyphenylamine)‐9,9′‐spirobifluorene (spiro‐OMeTAD), suffers from severe parasitic light absorption and moisture absorption.^[^ ^5^, ^6^ ^]^ By contrast, inverted PSCs could get rid of spiro‐OMeTAD and show advantages in stability and compatibility with the tandem structure. Therefore, inverted PSCs attracted more and more interest in recent years.

In inverted PSCs, fullerene and its derivatives are ideal ESLs. They can not only selectively extract electrons, but also passivate trap states in the perovskite layer.^[^ ^7^ ^]^ The bottlenecks of inverted PSCs mainly originate from the HSL, including poly(3,4‐ethylenedioxythiophene) polystyrene sulfonate (PEDOT: PSS), poly (triarylamine) (PTAA), NiO*_x_*, and so on. Because of the moisture absorption nature and relative shallow highest occupied molecular orbit (HOMO) level of PEDOT: PSS, corresponding PSCs generally exhibit modest efficiency and stability. PTAA could get rid of these shortcomings, but its poor wettability limits its compatibility with diverse solvents and perovskite compositions. What's worse, PTAA might be damaged by antisolvents, such as chlorobenzene (CB) and toluene, during the coating of the perovskite layer. NiO*_x_* possesses a hydrophilic surface and higher stability, while the adverse reactions between NiO*_x_* and perovskite are harmful to device performance. These materials have all propelled the development of inverted PSCs, but the ideal HSL remains to be found.

Self‐assembled monolayers (SAMs), which are generally composed of a terminal group, a linker group, and an anchoring group, provide a new way to construct HSLs.^[^ ^8^ ^]^ The interaction between the anchoring group and metal oxide (ITO, FTO, NiO*_x_*) underneath could tune the interfacial energy level to facilitate hole extraction. Compared with traditional HSLs based on *p*‐type semiconductors, SAMs exhibit the advantages of high transmittance, minimum parasitic absorption, and low series resistance. Moreover, the physical properties of SAMs, such as wettability, energy level, etc., could be adjusted by the linker and terminal groups to meet the requirements of PSC fabrication.^[^ ^9^ ^]^ Although SAMs have been studied for a long history, their flourishing in PSCs, especially inverted PSCs, started from the pioneering in 2018.^[^ ^10^, ^11^ ^]^ In this work, ─PO(OH)_2_ group‐based SAM was used as the HSL of PSC for the first time and exhibited great potential. In the next year, more advanced SAMs, namely (2‐(9H‐carbazol‐9‐yl)ethyl)phosphonic acid (2PACz) and [2‐(3,6‐Dimethoxy‐9H‐carbazol‐9‐yl)ethyl]phosphonic Acid (MeO‐2PACz), with carbazole group and ─PO(OH)_2_ group were reported. The corresponding PSCs overperformed PTAA‐based counterparts.^[^ ^12^ ^]^ From then on, SAM‐based PSCs attracted more and more interest.^[^ ^13^, ^14^ ^]^ However, SAMs also suffer from some shortcomings. First, it is rarely reported that SAMs could fully cover the substrate because of the insufficient anchoring sites on substrates, the steric hindrance between SAM molecules, and the aggregating tendency of SAM molecules.^[^ ^15^ ^]^ Second, the weak bonding between SAM and the metal oxide substrate or the perovskite might be detrimental to device stability.^[^ ^16^ ^]^ Finally, some desirable properties, such as hydrophilic surface and deep highest‐occupied‐molecular‐orbital (HOMO) level, are difficult to achieve simultaneously.^[^ ^17^ ^]^ In recent years, intensive efforts have been devoted to overcoming these obstacles while harvesting the benefits of SAMs in PSCs. For example, fabricating metal oxides with methods such as atomic layer deposition (ALD) or post‐deposition treatment methods (e.g., with UV and H_2_O_2_) could improve the anchoring‐site density on metal oxide substrates.^[^ ^18^, ^19^, ^20^ ^]^ Tuning the polarity of the solvent could suppress the aggregation of SAMs, thus benefitting the homogeneity.^[^ ^15^ ^]^ In addition, polymerizing the small SAM molecules into polymers was found to reduce their desorption from substrates, which significantly benefits the PSC stability.^[^ ^21^, ^22^ ^]^ Finally, tuning the molecular structure of the SAM could also bring about some fascinating properties.^[^ ^23^, ^24^, ^25^ ^]^ All these strategies were found to improve to a certain degree the performance of SAM‐based PSCs.

However, the reported SAM‐based HSLs are still not ideal because each SAM has its pros and cons, often leading to unavoidable trade‐offs. In this context, the combination of two kinds of SAMs, aka co‐SAM, opens a new era of SAM design and interface engineering. Co‐SAM is expected to show superiorities over the single SAM from several aspects, which include introducing additional functional groups to bond with the perovskite or the metal oxide substrate, reducing aggregation between SAM molecules via thermal dynamic regulation, and balancing the targets of hydrophilic surface and deep HOMO level. The enhanced interfacial contact could suppress the detachment during the working of PSCs. The reduced aggregation of SAM molecules could improve the coverage and reduce the undesirable current leakage. The hydrophilic surface of co‐SAM could benefit the growth of the perovskite film and reduce trap states at the interface. All these superiorities are favorable for the PSC's efficiency and stability under operational conditions.

**Figure 1:**
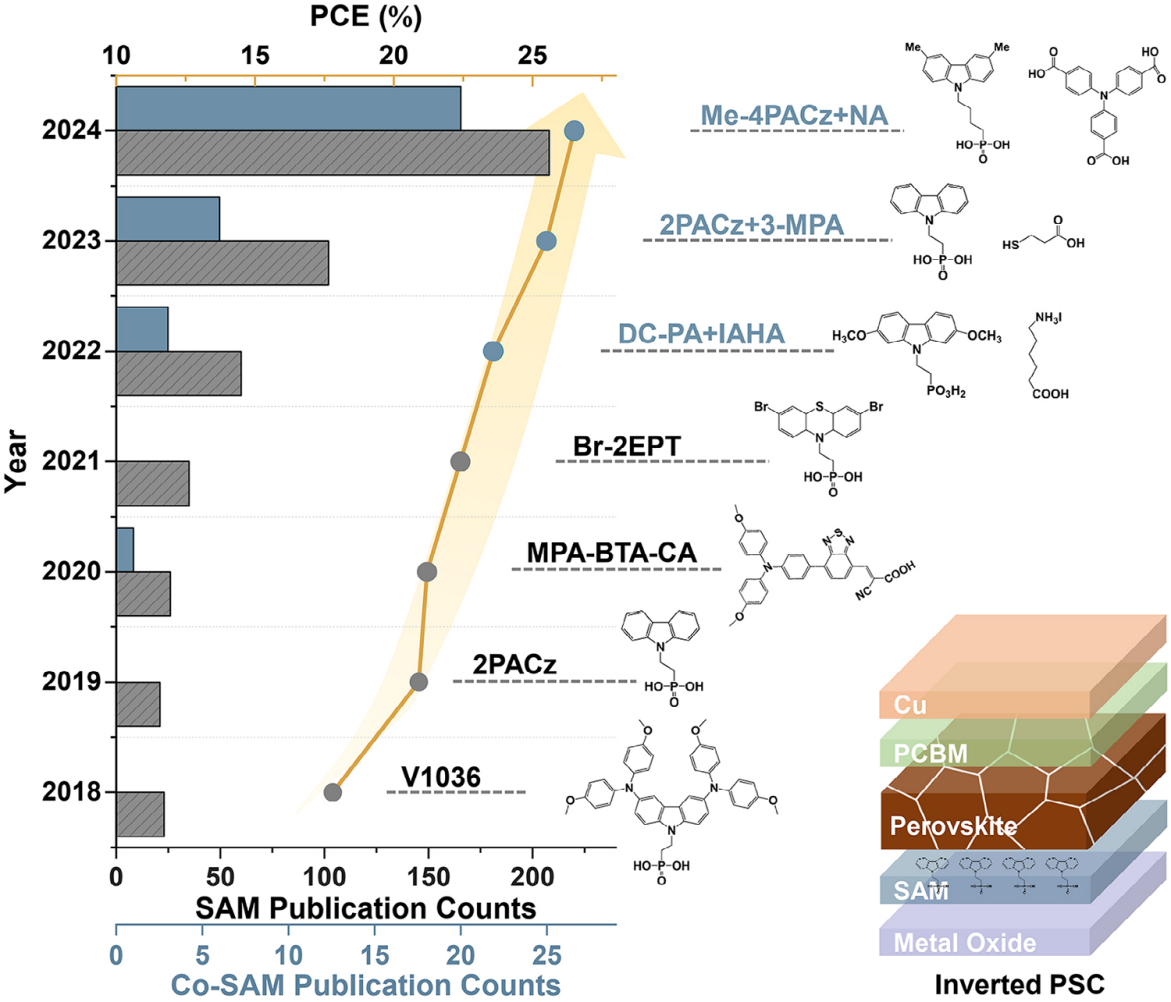
Annual counts of publications related to PSCs incorporating SAM or co‐SAM in the past seven years with data obtained from Web of Science on Jan. 27, 2025. Molecular structures of representative SAMs, corresponding PSC efficiencies, and the schematical structure of the inverted PSC are also shown.^[^ ^10^, ^12^, ^17^, ^27^, ^28^, ^29^, ^30^ ^]^

Regarding the recent research advancements concerning single SAMs in inverted perovskite single‐junction and tandem solar cells, we refer the readers to comprehensive reviews.^[^ ^26^ ^]^ **Figure** **1** exhibits the rapid development of SAM and co‐SAM‐based PSCs, including the count of publications and milestones of PCE improvement. The typical structure of inverted PSC and some representative molecular structures are also shown. **Table** **1** summarizes the photovoltaic performances of co‐SAM‐based PSCs. Although the benefits of engineering two SAMs have been recognized by a few works, there remain several open questions to be investigated. The insights provided in this perspective are hopefully expected to direct the future advancement of co‐SAM‐based PSCs.

**Table 1:** Photovoltaic parameters of co‐SAM‐based inverted PSCs, where *V*_oc_ refers to the open‐circuit voltage, *J*_sc_ refers to the short‐circuit current density, and FF refers to the fill factor.

SAMs	PVSK	*V*_oc_ [V]	*J*_sc_ [mA cm^−2^]	FF [%]	PCE [%]	Year	Refs.
Regular‐bandgap PSCs							
DC‐PA IAHA	Cs_0.05_MA_0.15_FA_0.80_PbI_3_	1.16	24.66	82.45	23.59	2022	[31]
Me‐4PACz MeO‐2PACz	Rb_0.05_Cs_0.05_MA_0.05_FA_0.85_Pb(I_0.95_Br_0.05_)_3_	1.18	25.58	84.9	25.58	2023	[32]
2PACz 3‐MPA	Cs_0.05_MA_0.1_FA_0.85_PbI_3_	1.16	25.9	84.2	25.3	2023	[29]
Me‐4PACz PC	(FA_0.98_MA_0.02_)_0.95_Cs_0.05_Pb(I_0.98_Br_0.02_)_3_	1.175	25.88	82.54	25.09	2024	[33]
MeO‐2PACz MPA	FA_0.82_MA_0.13_Cs_0.05_Pb(I_0.87_Br_0.13_)_3_	1.216	23.87	81.6	23.68	2024	[34]
Me‐4PACz MeO‐2PACz	Cs_0.175_FA_0.825_Pb(I_0.875_Br_0.125_)_3_	1.13	23.51	79.09	21.09	2024	[35]
2PACz MeO‐2PACz	(FA_0.98_MA_0.02_)_0.95_Cs_0.05_Pb(I_0.98_Br_0.02_)_3_	1.11	24.56	84.35	23.16	2024	[36]
Me‐4PACz Br‐EPA	Cs_0.05_(MA_0.05_FA_0.95_)_0.95_Pb(I_0.95_Br_0.05_)_3_	1.20	25.10	83.54	25.16	2024	[37]
2PACz PyCA‐3F	Cs_0.05_(FA_0.98_MA_0.02_)_0.95_Pb(I_0.98_Br_0.02_)_3_	1.17	25.21	86	25.37	2024	[38]
Me‐4PACz NA	FA_0.95_Cs_0.05_PbI_3_	1.192	26.47	84.11	26.54	2024	[30]
DMAcPA 4NPBA	(Cs_0.034_FA_0.966_)_0.99_MA_0.01_Pb(I_0.99_Br_0.01_)_3_	1.18	24.18	88	25.29	2024	[39]
MeO‐2PACz TBA	Cs_0.05_(FA_0.87_MA_0.13_)_0.95_Pb(I_0.9_Br_0.1_)_3_	1.185	23.63	83.21	23.31	2024	[40]
2PACz Me‐4PACz	Cs_0.05_FA_0.85_MA_0.1_PbI_3_	1.18	25.94	84.0	25.71	2024	[41]
2PACz TATPA	FA_0.84_MA_0.11_Cs_0.05_Pb(I_0.987_Br_0.013_)_3_	1.185	26.27	83.84	26.08	2025	[42]
MeO‐2PACz FC‐3283	Rb_0.05_Cs_0.05_MA_0.05_FA_0.85_Pb(I_0.95_Br_0.05_)_3_	1.19	25.91	83.50	25.70	2025	[43]
MeO‐2PACz Br‐4PADBC	Cs_0.05_MA_0.10_FA_0.85_PbI_3_	1.163	25.50	82.82	24.52	2025	[44]
4PADCB 4PBAI	Cs_0.05_(FA_0.95_MA_0.05_)_0.95_Pb(I_0.95_Br_0.05_)_3_	1.164	25.52	84.00	24.96	2025	[45]
Me‐4PACz MPTMS	FA_0.81_MA_0.07_Cs_0.12_PbI_3_	1.205	25.49	82.71	25.4	2025	[46]
Me‐4PACz FBA	FAMAPbI_3_	1.186	25.78	83.69	25.58	2025	[47]
CA‐Br TPA‐PT‐C6	MAPbI_3_	1.056	21.62	76.8	17.54	2020	[48]
Me‐4PACz 6dPA	FA_0.75_Cs_0.22_MA_0.03_Pb(I_0.82_Br_0.15_Cl_0.03_)_3_	/	/	/	20.9	2023	[49]
MeO‐2PACz MeIM	FA_0.9_MA_0.05_Cs_0.05_Pb(I_0.95_Br_0.05_)_3_	1.181	24.43	84.51	24.38	2024	[50]
MeO‐2PACz tBu‐4PACz	FA_0.84_MA_0.11_Cs_0.05_Pb(I_0.987_Br_0.013_)_3_	1.17	25.94	86.42	26.25	2024	[51]
MeO‐4PACz EA	FA_0.897_MA_0.047_Cs_0.055_PbI_2.858_Br_0.142_	1.169	24.94	83.76	24.42	2025	[52]
MeO‐2PACz CB‐PA	FA_0.95_Cs_0.05_PbI_3_	1.173	25.42	84.75	25.27	2025	[53]
Me‐4PACz DP	FA_0.95_Cs_0.05_PbI_3_	1.17	23.87	83.78	23.26	2025	[54]
2PACz TPAI	CsPbI_3_	1.26	20.74	82.95	21.60	2025	[55]
Narrow‐bandgap PSCs							
Br‐2PACz 4NH_3_CzI	Cs_0.25_FA_0.75_Pb_0.5_Sn_0.5_I_3_	0.819	31.61	75.0	19.45	2023	[56]
2PACz Glycine	FA_0.8_Cs_0.2_Sn_0.5_Pb_0.5_I_3_	0.884	33.17	80.76	23.46	2024	[57]
MeO‐2PACz 6PA	PEAFASnI_3_	0.829	17.6	64.5	9.4	2024	[58]
Wide‐bandgap PSCs and tandem PSCs							
Me‐4PACz CzTPA	FA_0.95_Cs_0.05_PbI_3_	1.172	25.76	84.92	25.66	2025	[59]
	FA_0.8_Cs_0.2_Pb(I_0.8_Br_0.2_)_3_	1.214	22.81	82.14	22.75		
Me‐4PACz Br‐4PACz	Cs_0.05_FA_0.85_MA_0.1_PbI_3_	1.187	25.79	85.82	26.28	2024	[60]
	Cs_0.2_FA_0.8_PbI_1.5_Br_1.5_	1.339	16.46	86.39	19.03		
Me‐4PACz MeO‐2PACz	FA_0.6_Cs_0.4_Pb(I_0.7_Br_0.3_)_3_	1.32	18.22	83.08	19.9	2023	[61]
2PACz MeO‐2PACz	Cs_0.2_FA_0.8_Pb(I_0.8_Br_0.2_)_3_	1.20	21.02	79.91	20.11	2023	[62]
2PACz Me‐4PACz	Cs_0.15_FA_0.65_MA_0.2_Pb(I_0.8_Br_0.2_)_3_	1.19	21.25	82.12	21.30	2024	[63]
Me‐4PACz MeO‐2PACz	Cs_0.05_FA_0.8_MA_0.15_ PbI_2.25_Br_0.75_	1.19	20.54	80.07	19.6	2024	[64]
Me‐4PACz MeO‐2PACz	FA_0.8_Cs_0.2_Pb(I_0.6_Br_0.4_)_3_	1.313	18.19	80.84	19.31	2024	[65]
Me‐4PACz 3‐APA	FA_0.75_Cs_0.25_Pb(I_0.8_Br_0.2_)_3_	1.255	85.5	20.88	22.4	2025	[66]
2PACz MeO‐2PACz	FA_0.7_MA_0.3_Pb_0.5_Sn_0.5_I_3_	1.22	16.5	80.6	16.2	2022	[17]
	Tandem	2.00	15.8	78.3	24.7		
Me4‐PACz PA	Cs_0.05_(FA_0.90_MA_0.10_)_0.95_Pb(I_0.80_Br_0.20_)_3_	1.23	21.15	85.25	22.24	2024	[67]
	Tandem	1.954	18.91	81.82	30.22		
2PACz Me‐4PACz	(FA_0.8_Cs_0.2_)Pb(I_0.6_Br_0.4_)_3_	1.32	16.90	81.62	18.25	2024	[68]
	Tandem	2.15	15.81	80.49	27.34		
Me‐4PACz MeO‐PhPACz	Cs_0.05_FA_0.8_MA_0.15_Pb(I_0.75_Br_0.25_)_3_	1.236	21.12	86.67	22.63	2024	[69]
	Tandem	1.893	18.54	79.97	28.07		
Me‐4PACz SA	FA_0.8_Cs_0.2_Pb(I_0.6_Br_0.4_)_3_	1.332	18.29	84.84	20.67	2025	[70]
	Tandem	2.146	16.19	83.29	28.94		
2PACz MeO‐2PACz	Tandem	1.86	19.4	79.6	28.3	2022	[71]

## 2 Deposition Methods

**Figure 2:**
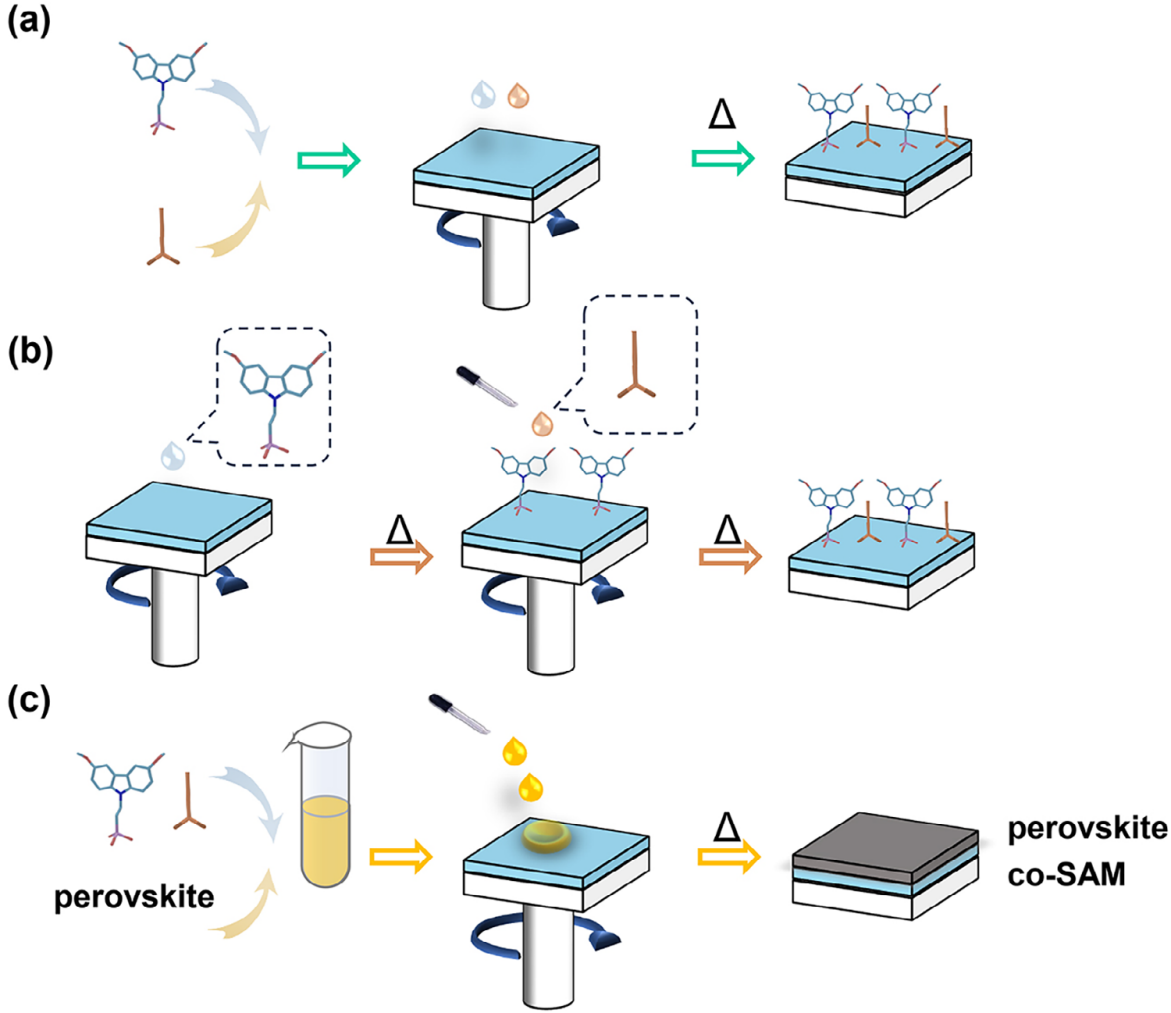
Schematical illustration of methods of preparing co‐SAM layers, including a) co‐adsorption, b) sequential deposition of co‐SAM, and c) deposition of co‐SAM via perovskite solution.

Regarding the deposition methods to prepare single SAMs, the readers are referred to the recent reviews by Yu et al. and Li et al.^[^ ^9^, ^26^ ^]^ As for co‐SAM, there have been three methods reported so far, namely co‐adsorption, sequential adsorption, and deposition simultaneously with perovskite. **Figure** **2** schematically illustrates these methods. Their key advantages are summarized in **Table** **2**.

**Table 2:** Comparison of various deposition methods of co‐SAM.

	Simple procedure	Improved wettability	Suppressed aggregation
Co‐adsorption	√		
Sequential deposition			√
Deposition with perovskite	√	√	

### 2.1 Co‐Adsorption

This is the simplest and most popular way to deposit co‐SAM. Two SAMs are dissolved in the solvent simultaneously and then coated onto substrates (Figure 2a). The properties of final co‐SAM depend on not only their respective physical and chemical properties but also the interaction and ratio between them.^[^ ^72^ ^]^ The rational design of co‐adsorbed co‐SAM for some desirable targets will be discussed in detail in the next section.

### 2.2 Sequential Deposition

The primary SAM is deposited onto a metal oxide substrate to form a loosely packed layer. Subsequently, the secondary SAM is deposited to fill the remaining exposed metal oxides (Figure 2b).^[^ ^37^, ^42^, ^68^ ^]^ Compared with the co‐adsorption strategy, sequential deposition avoids the undesirable segregation or interaction between different SAMs in the solution. Besides, the concentrations of SAM solutions are generally lower, which is favorable for the suppression of aggregation.

### 2.3 Deposition with Perovskite

SAMs could be dissolved in the perovskite precursor solution. Due to the relatively low solubility, SAM would precipitate earlier than perovskite and anchor onto ITO (Figure 2c).^[^ ^65^, ^73^ ^]^ In this way, the obstacle induced by the hydrophobic surface of SAM could be overcome, thus broadening the selection range of SAMs.

## 3 Design Principle

Owing to the additional functional groups and controllable interaction between SAMs, co‐SAM has delivered lots of superiorities. In this section, the design principles for various targets and relevant achievements are summarized.

### 3.1 Suppressing the Aggregation

As reported by Liu et al., amphiphilic SAM molecules (e.g., 2PACz) would aggregate into clusters. These clusters would be removed in the following washing step instead of anchoring on ITO, thus resulting in incomplete SAM and exposed ITO.^[^ ^15^ ^]^ To break up the aggregation of the primary SAM, the secondary molecule should be judiciously chosen to interact strongly with the primary SAM while avoiding any detrimental effect on the interfacial charge transport.

**Figure 3:**
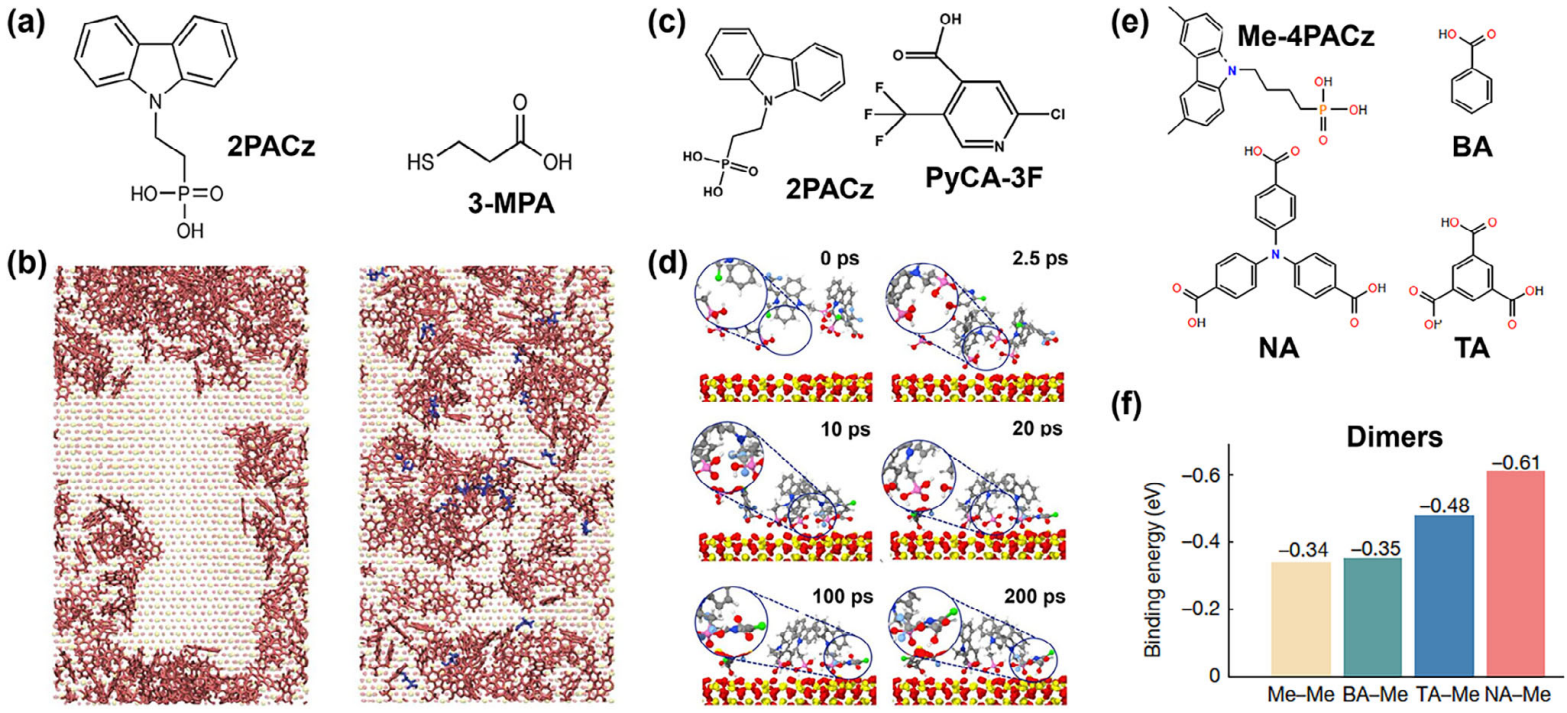
a) Molecular structures of 2PACz and 2‐MPA; b) simulated distribution of 2PACz and the mixture of 2PACz and 3‐MPA. (a,b) Adapted with permission.^[^ ^29^ ^]^ Copyright 2023, Springer Nature. c) Molecular structures of 2PACz and PyCA‐3F; d) simulated structural evolution of the mixture of 2PACz and PyCA‐3F. (c,d) Adapted with permission.^[^ ^38^ ^]^ Copyright 2024, Springer Nature under a Creative Commons Attribution 4.0 International License (http://creativecommons.org/licenses/by/4.0/). e) Molecular structures of Me‐4PACz, BA, NA, and TA; f) binding energy of various dimers. (e,f) Reproduced with permission.^[^ ^30^ ^]^ Copyright 2024, Springer Nature.

In a pioneering attempt to suppress the SAM aggregation using the co‐SAM strategy, Park et al. introduced 3‐mercaptopropionic acid (3‐MPA), which contains a thiol group (─SH) and a carboxyl group (─COOH), to combine with the widely used 2PACz.^[^ ^29^ ^]^ The ─SH group could interact with the ─PO(OH)_2_ group in 2PACz to break apart their cluster while the ─COOH group could also anchor on substrates (**Figure** **3**a). Similarly, Li et al. combined 2‐chloro‐5‐(trifluoromethyl)isonicotinic acid (PyCA‐3F) with the widely used 2PACz.^[^ ^38^ ^]^ The trifluoromethyl groups (─CF_3_) in PyCA‐3F could coordinate with the ─PO(OH)_2_ groups in 2PACz through hydrogen bonds. Such a reaction restricts the mobility of 2PACz and mitigates the formation of clusters, which has been verified to be effective in both the adsorption and sequential deposition methods (Figure 3b). Furthermore, Liu et al. adopt benzoic acid (BA), trimesic acid (TA), and 4,4′,4″‐nitrilotribenzoic acid (NA) to break up [4‐(3,6‐dimethyl‐9H‐carbazole‐9‐yl)butyl] phosphonic acid (Me‐4PACz) clusters and compared their effects via the joint simulation and experimental methods.^[^ ^23^ ^]^ They discovered that NA showed the most reaction with Me‐4PACz among three cluster breakers. The binding energy between NA and Me‐4PACz is even comparable with that of Me‐4PACz trimers. It indicates that the reaction between NA and Me‐4PACz could compete over the formation of Me‐4PACz tetramers, thus reaching the target of breaking up clusters (Figure 3c). Based on the co‐SAM composed of NA and Me‐4PACz, the inverted PSC achieved a certified steady‐state PCE of 26.54%, which is to date the highest published value for PSCs. Moreover, the mini‐module with an aperture area of 11.1 cm^2^ achieved a certified PCE of 22.74%, indicating that this strategy is compatible with large‐scale manufacturing.

In all the works above, the primary SAM contains a ─PO(OH)_2_ group to anchor onto the metal oxide surfaces and provides the interfacial dipoles to facilitate hole transport. As a general principle, the secondary SAM molecule should interact with the ─PO(OH)_2_ group to compete over the aggregation process and enable the uniform dispersion of the primary SAM. It is interesting to note that in all these publications the secondary SAM contains the ─COOH group to anchor on the metal oxide substrate.^[^ ^40^, ^59^ ^]^ As demonstrated in the ground‐breaking works mentioned above, the co‐SAM with improved coverage and homogeneity has been obtained successfully, which led to enhanced photovoltaic performance.^[^ ^46^, ^55^ ^]^

### 3.2 Enhancing the Interfacial Contact

**Figure 4:**
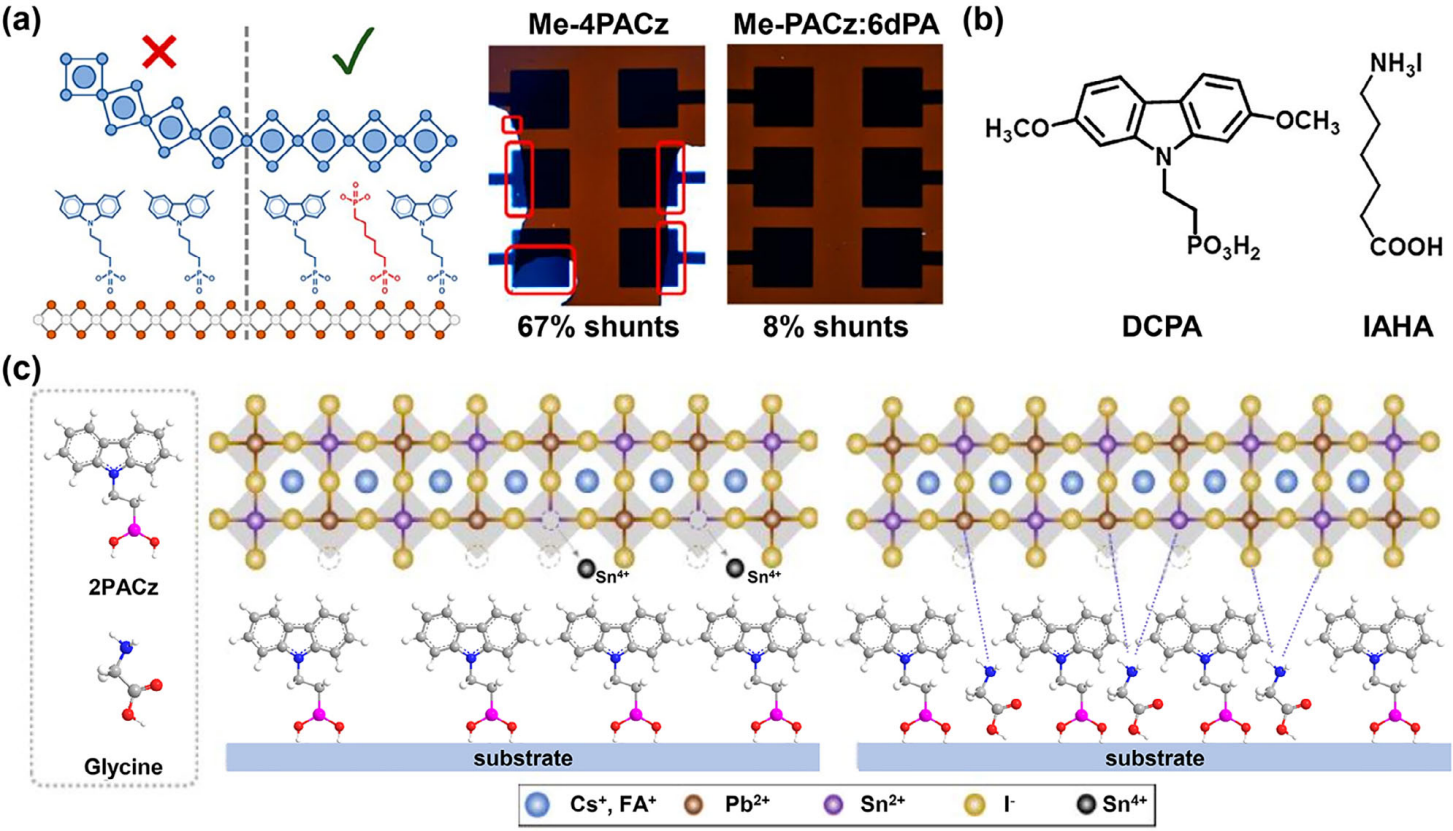
a) Schematic illustration of interfacial structures of Me‐4PACz/perovskite and Me‐4PACz:6dPA/perovskite as well as photographs of corresponding PSCs. Reproduced with permission.^[^ ^49^ ^]^ Copyright 2023, American Chemical Society. b) Molecular structures of DCPA and IAHA. Reproduced with permission.^[^ ^31^ ^]^ Copyright 2022, Wiley‐VCH. c) The lattice structure of Sn─Pb perovskite films coated on ITO/2PACz and ITO/2PACz: glycine. Adapted with permission.^[^ ^57^ ^]^ Copyright 2024, American Chemical Society.

The widely used SAMs, e.g. 2PACz and Me‐4PACz, generally show poor wettability, which seriously hinders the adhesion of perovskite precursor solutions. The introduction of secondary SAM with the hydrophilic group could overcome this obstacle. For example, Al‐Ashouri et al. added 1,6‐hexylenediphosphonic acid (6dPA) into Me‐4PACz for improved wettability. In this way, shunt pixels of perovskite film decreased from 67% to 8% (**Figure** **4**a). The reproducibility of PSCs was significantly improved, which is vital for large‐scale manufacturing in the future.^[^ ^49^ ^]^ Some others use MeO‐2PACz with hydrophilic ─OCH_3_ group as the secondary SAM and obtained similar results, thus confirming the reliability and universality of this design principle.^[^ ^32^, ^35^, ^36^, ^39^, ^43^, ^51^, ^53^, ^61^, ^62^, ^64^, ^67^, ^71^ ^]^

The secondary SAM could also introduce functional groups to bond with perovskite.^[^ ^48^, ^50^, ^52^, ^54^, ^70^ ^]^ For example, Deng et al. mixed ((2,7‐dimethoxy‐9*H*‐carbazol‐9‐yl) methyl) phosphonic acid (DCPA) and 6‐(iodo‐λ^5^‐azanyl) hexanoic acid (IAHA) to fabricate the co‐SAM. As shown in Figure 4b, IAHA is a bit longer than DCPA, Therefore, its ammonium group can be exposed to interact with perovskite. The enhanced interfacial contact led to improved efficiency and stability of PSCs.^[^ ^31^, ^33^, ^39^, ^42^, ^47^, ^58^, ^68^, ^74^ ^]^ Roe et al. reported the co‐SAM consisted of 2PACz and glycine. The amine (─NH_2_) of glycine and ammonium (─NH_3_^+^) groups generated after protonation can effectively passivate Sn^4+^ defects at the buried interface of Sn─Pb perovskite films (Figure 4c). Consequently, the co‐SAM‐based PSC delivered an efficiency of 23.46%, which outperformed PSCs using 2PACz or glycine alone.^[^ ^57^ ^]^ Dong et al. reported a self‐assembled bilayer by covalently interconnecting triphenylamine onto the top of 2PACz, resulting in the polymerized network with face‐on orientation to connect perovskite. The enhanced interfacial adhesion led to significantly improved hole extraction and thermal stability.^[^ ^42^ ^]^ Using the covalently interconnected co‐SAM, the PSC achieved an efficiency exceeding 26% and demonstrated less than 4% and 3% efficiency loss after 2000 h damp heat exposure (85 °C and 85% relative humidity) and over 1200 thermal cycles between −40 °C and 85 °C, respectively, meeting the temperature stability criteria outlined in the International Electrotechnical Commission 61 215:2021 standards.

### 3.3 Energy‐Level Alignment

**Figure 5:**
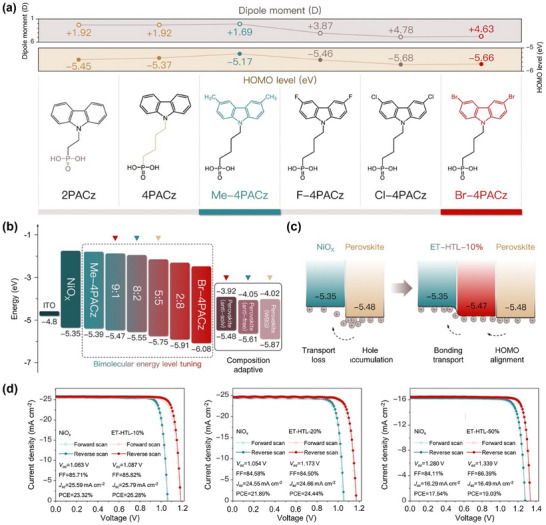
a) The dipole moment value, HOMO level, and molecular structure of various SAMs; b) energy‐level diagram of the PSCs with various co‐SAM and various perovskite layers; c) hole‐transport diagram at the NiO*_x_*/perovskite and co‐SAM/perovskite interfaces; d) JV curves of various PSCs with and without the co‐SAM. (a–d) Adapted with permission.^[^ ^60^ ^]^ Copyright 2024, The Royal Society of Chemistry.

Perovskites refer to a family of materials, whose bandgap can be easily tuned by the composition. Therefore, PSCs with narrow bandgap (≈1.2 eV), middle bandgap (≈1.5 eV), and wide bandgap (≈1.7 eV) could all be achieved. A specific SAM can hardly form a good energy‐level alignment with various perovskites. The combination of two SAMs with different dipole moments is able to continuously tune the HOMO level.^[^ ^44^, ^45^, ^66^ ^]^ Peng et al. selected two SAMs with different dipole moments (Me‐4PACz and [4‐(3,6‐dibromo‐9H‐carbazole‐9‐yl)butyl] phosphonic acid (Br‐4PACz)) to construct co‐SAM (**Figure** **5**a). Through varying their ratio, the HOMO level of the co‐SAM was tuned from −5.39 to −6.08 eV (Figure 5b). Using the co‐SAM with a specifically optimized ratio, three PSCs with bandgaps of −5.48, −5.61, and −5.87 eV all achieved high performance, confirming the success of energy‐level tuning by co‐SAM (Figure 5c).^[^ ^60^ ^]^ Torres Merino et al. used a similar strategy to tune the HOMO level of the co‐SAM for various wide‐bandgap PSCs. They elucidate that the matched energy level at the co‐SAM/perovskite interface could suppress charge accumulation, and then suppress halide segregation in the wide‐bandgap perovskite, which is favorable for the device stability under the operational condition.^[^ ^75^ ^]^

## 4 Summary and Outlook

In this perspective, we summarized the latest achievements in deposition techniques, molecule design, working principle, and application of co‐SAM in PSCs. Starting from the molecule design of SAMs, we discussed their functional groups, and subtle factors such as steric hindrance may exert great influence on their film quality and functionalities as the interface mediator. Then the deposition techniques and working principles of co‐SAM were explained, which offer valuable insights into the self‐assembling process of these molecules, and in fact, the interaction mechanisms between molecules during this process necessitate the development of co‐SAM and other designs. Lastly, we summarize that co‐SAM can be successfully applied to various interfaces, presenting merits such as tunable energy level and synergetic materials combination.

Despite the significant advancements in the past few years, several challenges about SAM and co‐SAM still need to be tackled. Future studies may be directed toward, but not limited to, the following directions.

1. Mechanism exploration: Some basic properties of co‐SAM remain mysterious and worthy of further investigation. In the clusters of SAMs, whether the anchoring group was exposed to the surface or wrapped inside remains controversial.^[^ ^15^, ^29^, ^30^ ^]^ The direct observation of the aggregation process has not been realized yet. In‐depth characterization techniques, such as highly sensitive optical measurements, transmission electron microscopy (TEM), and scanning tunneling microscope (STM) may offer valuable insights into the atomic structure of the SAM/co‐SAM layer. Previously, Liu et al. adopted TEM to observe SAM micelles and measured their sizes.^[^ ^15^ ^]^ S. Park used high‐angle annular dark‐field (HAADF) scanning transmission electron microscopy (STEM) to exhibit the homogeneity of the SAM and co‐SAM.^[^ ^29^ ^]^ Franz et al. demonstrated by STM the formation of NHC monolayers on Si (111) using different model NHCs, and the direct bonding leads to high thermal stability.^[^ ^76^ ^]^ We believe these techniques are poised to provide more insights and open new research avenues in the photovoltaic field. Direct observation of the dynamic assembling process may shed light on the interaction characteristics between co‐adsorbed molecules, providing quantitative information on intra‐molecule covalent bonds, hydrogen bonds, etc. Understanding these nanostructures and interactions can benefit the design of better materials with tailored properties.
2. Materials design: Exploring novel SAMs with tailored sizes, configurations or functional groups remains a crucial and promising research thrust. Nowadays, almost all successful SAM materials rely on the ─PO(OH)_2_ anchoring group. If new SAMs with other anchoring groups could lead to high‐performance PSCs, the design of co‐SAM will have more choices, and the larger design space may result in more fascinating properties. For example, the SAM with a boric acid anchoring group could overcome the corrosive effect induced by ─PO(OH)_2_ while maintaining excellent PSC performance.^[^ ^77^ ^]^ As for the terminal group, the benzene ring with intermolecular *π*–*π* interactions can alter the stacking mode of SAMs toward tight assembling and face‐on orientation.^[^ ^25^ ^]^ As for the linking group, phenylene can provide higher hole‐transport capability compared with the carbon chain.^[^ ^23^ ^]^ The co‐SAM flexibly utilizing these characteristics has the potential to further improve the performance of PSCs from multiple aspects. Another equally important route to optimizing co‐SAM is using new deposition techniques, for example entailing thermal evaporation and spray coating, which may induce different growing dynamics and nanostructures.
3. Application extension: The tunable energy levels and improved homogeneity of co‐SAM could also benefit other optoelectronic devices. For example, in perovskite light‐emitting diodes (PeLEDs), red, green, and blue emission layers use different perovskite materials, which raises the requirement for different hole inject materials. Equipped with a vast design space, co‐SAM may be able to meet this requirement. Co‐SAM‐enabled applications may also be extended to photodetectors, field effect transistors, and other domains. Nowadays, most perovskite‐based optoelectronic devices are fabricated in laboratories in small areas with poor scalability. In the forthcoming industrialization process, it is essential to fabricate all functional layers over a large area with a high homogeneity. The principle of co‐SAM suppressing aggregation can be applied to large‐area deposition processes such as blade coating and slot die coating, to achieve large‐area and homogeneous HSL.^[^ ^30^ ^]^ As the first layer in the device, co‐SAM lays a solid foundation for the deposition of the following layers. The new height of co‐SAM design and synthesis reached in inverted PSCs opens new opportunities for the advancement and commercialization of a diverse range of technologies with high performance and reliability.

## Conflict of Interest

The authors declare no conflict of interest.
